# Evaluating *Protoparvovirus carnivoran1* Risk in Wild Carnivorans and Hunting Dogs in the Valencian Community, Eastern Spain

**DOI:** 10.1155/tbed/6899927

**Published:** 2026-07-28

**Authors:** Francesca Suita, Alba Martí-Marco, Víctor Lizana, Jordi López-Ramon, João Torres da Silva, Jesús Cardells

**Affiliations:** ^1^ Service for Analysis, Research, and Management of Wild Animals (SAIGAS), Veterinary Faculty, Cardenal Herrera-CEU University, CEU Universities, Valencia 46115, Spain, uchceu.es; ^2^ Vaersa (Valencian Waste Energy Utilization S.A.), Generalitat Valenciana, Valencia, Spain, gva.es

**Keywords:** canine parvovirus, feline panleukopenia virus, molecular detection, *Protoparvovirus carnivoran1*, relative risk mapping, risk factors, sequencing

## Abstract

*Protoparvovirus carnivoran1* is a highly contagious pathogen that poses a significant threat to both domestic and wild carnivorans. Despite its importance, the dynamics of viral transmission at the wildlife–domestic interface remain poorly understood. Between 2017 and 2025, 303 fecal samples from eight free‐ranging mesocarnivoran species and 243 samples from hunting dogs (*Canis lupus familiaris*) were collected in the Valencian Community. For the dog cohort, surveys were conducted to evaluate potential risk factors for infection. All samples were screened via real‐time PCR, and positive cases were characterized by sequencing key VP2 regions. *P. carnivoran1* DNA was detected in 11.11% (27/243) of dogs and 2.6% (8/303) of mesocarnivorans. Among wildlife, the red fox (*Vulpes vulpes*) was the most affected (*n* = 3), followed by beech martens (*Martes foina*), Eurasian badgers (*Meles meles*), and a common genet (*Genetta genetta*). Multivariable analysis identified a strong statistical association between raw meat intake and CPV‐2 positivity (*p*  < 0.001). Furthermore, spatial analysis through relative risk (RR) maps identified distinct geographical clusters of infection, suggesting localized areas of higher environmental viral load. Sequencing revealed that all dog‐derived strains clustered within the CPV‐2c antigenic variant, whereas mesocarnivorans harbored predominantly feline panleukopenia virus (FPV)‐like strains (*n* = 6), with only two individuals infected with CPV‐2b. Phylogenetic analysis showed a predominance of the “Asian” lineage (324I, 370R) among hunting dogs, while wild carnivorans exhibited a more varied distribution, including European‐like and Asian‐related signatures (324I). These findings highlight differences in the viral strains detected across these populations, which may reflect distinct epidemiological dynamics in the region. Further surveillance is needed to better define the extent of viral circulation and the ecological drivers at the wildlife–domestic interface.

## 1. Introduction

The species *Protoparvovirus carnivoran1* (genus *Protoparvovirus*) includes several antigenically and genetically closely related viruses that cause significant disease in carnivorans [1]. Key members of this lineage include feline panleukopenia virus (FPV) and canine parvovirus type 2 (CPV‐2). While feline panleukopenia was clinically described as early as the 1920s [2], the virus itself was first isolated in the 1960s [3]. FPV has since been documented in domestic cats and various large felids, including tigers (*Panthera tigris*), cheetahs (*Acinonyx jubatus*), and lions (*Panthera leo*) [4]. In contrast, CPV‐2 emerged in the late 1970s through the adaptation of an FPV‐like precursor circulating in wild carnivorans [5] and rapidly achieved worldwide endemic distribution in domestic dog populations [4]. Following its initial emergence, the original CPV‐2 type underwent rapid diversification; the CPV‐2a variant largely replaced the original type, followed by the appearance of CPV‐2b and CPV‐2c, all of which currently cocirculate globally [6]. CPV‐2c, defined by a glutamic acid substitution (Asn/Asp426Glu) at residue 426 of the VP2 capsid protein, was first detected in Italy in 2000 [7]. Additionally, the continuous evolution of CPV‐2 has led to the emergence of diverse lineages with distinct geographical patterns, including the recent global expansion of “Asian‐like” variants, which may complicate the epidemiological landscape at the wildlife–domestic interface [8].

The emergence of CPV‐2a and subsequent variants (CPV‐2b and CPV‐2c) was associated with an expanded host range, including the ability to infect felids, a trait absent in the original CPV‐2 type [9]. Notably, some studies have reported a higher prevalence of CPV‐2a/2b infections in large felids compared to domestic cats [10]. However, this trend requires a nuanced interpretation: although it may suggest increased biological susceptibility, it could also reflect heightened environmental exposure to canine strains in areas where domestic and wild populations overlap. This exposure is particularly concerning given that the current antigenic variants (CPV‐2a, CPV‐2b, and CPV‐2c) are reported to cause more severe disease, be shed in the feces at higher titers—and in the case of CPV‐2c, for a prolonged time—and require a lower infectious dose [11]. Significantly, recent evidence suggests that the CPV‐2c variant plays a critical role in the virus’s transmission across an even broader host spectrum, extending from various carnivorans to even‐toed ungulates and pangolins [12].

CPV infection commonly causes a highly contagious disease primarily transmitted via the fecal‐oral route [13]. While direct contact is a highly efficient means of transmission, this nonenveloped virus is exceptionally resistant and can stay infectious in the environment for months and possibly years [14–16]. Consequently, indirect transmission through contaminated fomites represents a significant means of infection [13], as direct contact between hosts is not strictly required for the virus to spread efficiently [14]. This remarkable environmental stability, combined with the movement of infected animals, is a key factor in the virus’s ability to persist and disperse across wide geographical areas [15].

In the context of the wildlife‐domestic interface, initial understanding suggested that infections in wildlife were largely “spillovers” from the domestic cycle [17]. However, molecular studies have established that wild and domestic carnivorans often share identical or closely related *P. carnivoran1* variants, confirming transmission across this interface [18]. Consequently, wild hosts are now understood to serve as virus reservoirs and potential sources of infection for the domestic animal population [17]. Despite widespread domestic vaccination, FPV and CPV‐2 remain important pathogens [15]. Disease severity varies greatly depending on the host species, age, immunity, and viral strain. Infection in young domestic carnivorans and large felids can range from subclinical to lethal, typically presenting with leukopenia (and therefore affecting the immune response), fever, and severe gastroenteritis [12, 14]. In captive and wild felines, the pathogenicity of recently emerged variants like CPV‐2c appears similar to FPV, potentially spreading more efficiently in these populations [19, 20]. Furthermore, epidemic waves can cause significant declines in naïve wild populations, as observed in North American gray wolves (*Canis lupus*) [20], Ethiopian wolves (*Canis simensis*) [21], and more recently in small Indian civets (*Viverricula indica*) [22]. Given the importance of infectious diseases in carnivoran conservation [23] and the difficulty of assessing health in wild litters, the true impact of *P. carnivoran1* in wild ecosystems remains poorly understood [18], especially for immunologically naïve reintroduced animals, like the Iberian lynx (*Lynx pardinus*) [24].

Molecular surveillance of *P. carnivoran1* in Spanish wild carnivorans confirms their endemic and widespread distribution, with varying prevalence levels reported across multiple regions: the west (Extremadura) in Iberian lynx (*L. pardinus*) [25]; the center (Castilla‐La Mancha) in Iberian lynxes and domestic dogs [24]; and the north (Navarra and surrounding areas) across various host species [18, 26]. Serological data further indicate widespread exposure in wildlife populations across central and northern Spain [27–31]. However, the Valencian Community (Eastern Spain) currently represents a significant knowledge gap, as the circulation and genetic diversity of *P. carnivoran1* in this region remain uncharacterized. Geographically, the Valencian Community lies within the latitude band (39°N) that has been globally identified as a zone of high potential risk for canine *P. carnivoran1* distribution, due to its favorable bioclimatic variables [32]. Furthermore, the high density of the registered canine population, estimated at 979,411 dogs in 2024 [33] in rural and urban areas of the Valencian Community, combined with potential challenges regarding vaccination coverage, contributes significantly to maintaining the endemic risk [34]. Therefore, the primary aim of this study was to assess viral prevalence, genetic diversity, and associated infection risk in both mesocarnivorans and a key domestic representative (hunting dogs) within this region. Hunting dogs were specifically selected due to their ecological proximity to wildlife, which positions them as crucial agents for viral transmission and environmental contamination.

## 2. Materials and Methods

### 2.1. Study Area and Sample Collection Strategy

**Figure 1:**
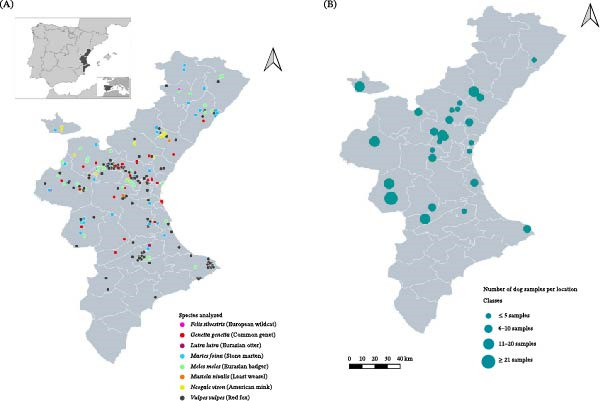
Geographic distribution of study samples in the Valencian Community, Spain. (A) Collection locations of wild carnivorans (*n* = 303); individual markers represent specific detection points with colors indicating identified species. (B) Spatial distribution and sampling intensity of hunting dogs across the 28 sampled municipalities. Dot size in subfigure (B) corresponds to the sample size collected at each location, ranging from ≤5 to ≥21 individuals. In both subfigures, white lines indicate county boundaries. The inset maps in (A) show the location of the Valencian Community within the Iberian Peninsula and the Peninsula’s position within Europe.

The study was conducted within the Valencian Community, an autonomous region situated on the eastern coast of Spain. Given this broad geographical scope, sampling efforts focused on two distinct populations: wild carnivorans collected through passive surveillance and domestic hunting dogs, whose sampling areas covered the three provinces of the community, as detailed in Figure 1.

#### 2.1.1. Wild Carnivoran Sampling

Between February 2017 and November 2025, a total of 303 wild carnivoran fecal samples were collected across the Valencian Community (Figure 1). The sampled cohort included individuals belonging to eight species across four families: 158 red foxes (*Vulpes vulpes*; Canidae); 45 Eurasian badgers (*Meles meles*), 42 beech martens (*Martes foina*), 2 Eurasian otters (*Lutra lutra*), 2 least weasels (*Mustela nivalis*), and 22 American minks (*Neogale vison*) (Mustelidae); 4 European wildcats (*Felis silvestris*; Felidae); and 28 common genets (*Genetta genetta*; Viverridae).

Most of the specimens (211/303) were obtained as roadkill. Additional sources included 31 red foxes obtained through authorized hunting events and 21 American minks recovered from official eradication campaigns targeting this invasive species. The remaining samples (40) were provided by local authorities and the “La Granja” Wildlife Rescue Center. This latter group included animals found dead from natural causes (three red foxes and one wildcat), those illegally trapped (three common genets, one red fox, and one Eurasian badger), those that died from electrocution (six beech martens and five common genets), and seven individuals that were brought to the wildlife center for medical reasons, sampled during treatment, and subsequently released (three beech martens, two red foxes, one least weasel, and one Eurasian otter). The cause of death remained unspecified for 13 individuals (10 red foxes, two Eurasian badgers, and one wild cat).

All wild carnivoran carcasses underwent a thorough macroscopic examination to assess the external and internal lesions. During the examination, fecal samples were collected aseptically from the large intestine and stored individually at −20°C until further processing.

#### 2.1.2. Hunting Dog Sampling and Survey

The canine sampling was conducted randomly across 28 municipalities within 18 different districts of the Valencian Community (Figure 1). This phase lasted 1 year, from February 2021 to February 2022, and involved collecting fecal samples from 243 hunting dogs. Detailed general information (location, sex, age, and breed) and specific risk factors were recorded for each individual. The risk factors assessed included dog ownership (private owner vs. pack), housing type (individual vs. collective), and the total number of dogs in the collective. Furthermore, the hunting modality practiced (big and/or small game) and hunting area (within the Valencian Community and/or outside), ground substrate in the kennels (natural soil or concrete), water source (tap, well, and/or natural), type of diet (commercial/dry feed, homemade cooked, and/or homemade untreated, meaning raw meat and offal from hunted animals), other behaviors (coprophagia, scavenging, and/or ingestion of waste), and an assessment of fecal consistency based on the Bristol stool scale [35] were also evaluated.

Fecal samples from domestic dogs were collected as freshly voided stools using an aseptic protocol and stored at −20°C, ensuring consistency with the processing and storage of samples obtained from wild carnivorans.

### 2.2. Molecular Detection of *P.carnivoran1*

DNA was extracted from ~25 mg of feces using the NZY Tissue gDNA Isolation Kit (NZYTech, Lisboa, Portugal), following the manufacturer’s instructions. DNA was eluted in 60 μL of elution buffer and stored at −20°C until real‐time PCR analysis. DNA concentration was not measured, and samples were not diluted to equalize the DNA concentration prior to real‐time PCR.

Detection of *P. carnivoran1* DNA was carried out following the method described by Streck et al. [36] by amplification of a 200 bp fragment of a conserved region of the VP2 gene using a TaqMan‐based real‐time PCR. Each 20 μL reaction contained 10 μL of NZYSpeedy qPCR Probe Master Mix (2×) with ROX (NZYTech), 8 pmol of each primer (forward: 5^′^‐TGGAACTAGTGGCACACCAA‐3^′^; reverse: 5^′^‐AAATGGTGGTAAGCCCAATG‐3^′^), 4 pmol of the TaqMan probe (FAM‐CAGGTGATGAATTTGCTACAGG‐BBQ), and 2 μL of template DNA.

Reactions were run on a QuantStudio 5 system (Applied Biosystems, Waltham, MA, USA) with the following cycling protocol: initial denaturation at 95°C, followed by 40 cycles of denaturation at 95°C, and annealing and extension at 60°C. A no‐template control was included in each run as a negative control. As a positive control, DNA extracted from a fecal sample of a kitten showing clinical signs compatible with FPV infection and a positive ELISA result (SNAP Parvo, Idexx, Westbrook, ME, USA) was used. This commercial ELISA, though designed for CPV, has been conclusively shown to reliably detect FPV in clinical feline samples [37].

Samples were considered positive when they showed a quantification cycle (Cq) value < 37, a Cq confidence ≥ 3.0, and an amplification score ≥ 0.7. The fluorescence threshold was set at 0.2. Borderline or doubtful results were retested for confirmation.

### 2.3. Sequencing

To genetically characterize the *P. carnivoran1*‐positive samples, three distinct regions of the VP2 gene were partially sequenced using Sanger sequencing (Macrogen Genomics, Spain). These regions were selected because they encompass key residues for viral characterization, including 80, 87, 93, and 103, as well as residues 297, 300, 305, 323, and 426. Together, these positions are critical for differentiating FPV from CPV‐2 and for identifying specific CPV‐2 variants (CPV‐2a, 2b, and 2c), as previously described [4, 38]. While the first fragment was sequenced separately, the second and third regions were designed to overlap, ensuring continuous coverage of key molecular markers. The first fragment (“region 1”) targeted the 5^′^ end of the gene (nt 214–434) using M1 and M2 primers. A central portion (“region 2,” nt 820–1324) was analyzed using the CPV‐3F and CPV‐3R sets. Finally, a third fragment (‘region 3’) was amplified to encompass codon 426, which is critical for CPV‐2 variant discrimination. For this region, two primer sets were employed: the 555 for/555rev primers (nt 1268–1850) and a newly designed more compact pair (VP2_426F/R), developed using the NCBI Primer‐BLAST tool [39], covering nt 1216–1454. These additional primers were utilized to optimize amplification success in hunting dog fecal samples as shorter amplicons (239 bp versus 583 bp) are less susceptible to DNA degradation and PCR inhibitors frequently encountered in field‐collected specimens. Primer pairs used in this study are detailed in Table 1.

**Table 1:** Oligonucleotide primers used for partial amplification and sequencing of the *Protoparvovirus carnivoran1* VP2 gene.

Primers	Nucleotide sequence (5^′^→3^′^)	Position (nt)	Thermal cycling profile	References
M1 M2	GAAAACGGATGGGTGGAAAT AGTTGCCAATCTCCTGGATT	214–233 415–434	95°C for 2 min; 40 cycles of: 95°C for 5 s, 52°C for 30 s	[40]
CPV‐3F CPV‐3R	ACAGGTGATGAATTTGCTACAG TTACAGGAAGGTTAAAGTTAAT	820–841 1303–1324	95°C for 2 min; 40 cycles of: 95°C for 5 s, 55°C for 30 s	[4]
555for 555rev	CAGGAAGATATCCAGAAGGA GGTGCTAGTTGATATGTAATAAACA	1268–1287 1826–1850	95°C for 2 min; 40 cycles of: 95°C for 5 s, 55°C for 30 s	[7]
VP2_426F VP2_426R	ACCACAACAGGAGAAACACCT TGACCATTTGGATAAACTGGTGG	1216–1236 1432–1454	95°C for 2 min; 40 cycles of: 95°C for 5 s, 61°C for 30 s	This study

*Note:* This table specifies the nucleotide sequence (5^′^→3^′^), the corresponding genomic position based on the VP2 reference sequence EF418569.1 (GenBank accession number), the specific thermal cycling profile, and the original publication for each primer pair.

Real‐time PCR amplifications for sequencing were performed on a QuantStudio 5 system (Applied Biosystems, Waltham, MA, USA) in 20 μL reactions using 10 μL of NZYSpeedy qPCR Green Master Mix (2×) (NZYTech, Lisbon, Portugal). Due to the kinetic optimization of this master mix, a two‐step cycling protocol was implemented. The conditions included an initial denaturation at 95°C for 2 min, followed by 40 cycles of denaturation at 95°C for 5 s, and a combined annealing/extension step at primer‐specific temperatures (Table 1) for 30 s.

### 2.4. Phylogenetic Analysis

Phylogenetic analysis of the identified parvovirus isolates was inferred using the maximum likelihood (ML) method based on a partial fragment of the *VP2* gene (~400 bp) and reference sequences from domestic dogs and wild carnivores retrieved from the GenBank database (accessed on 17 June 2026). Multiple‐sequence alignment was performed using the ClustalW algorithm in MEGA v12 software (Molecular Evolutionary Genetics Analysis, Temple University, Philadelphia, PA, USA) [41]. Prior to tree construction, the best‐fit nucleotide substitution model was determined using the Bayesian Information Criterion (BIC) in MEGA v12. The Tamura 3‐parameter (T92) model was selected, incorporating a gamma distribution (+G) to account for evolutionary rate differences among sites. Branch reliability was assessed via bootstrap analysis with 1000 replicates. Gaps and missing data were handled using partial deletion with a 95% site coverage cutoff. To establish evolutionary directionality, the tree was rooted using an ancestral FPV sequence from 1985 (M10824.1). For final visualization, only bootstrap values ≥50% were displayed on the nodes to ensure the clarity of the phylogenetic relationships.

### 2.5. Statistical Analysis

Prevalence rates and corresponding 95% confidence intervals (CI) were calculated for the overall study population and stratified by host species and population. For wild mesocarnivorans, due to the low number of positive outcomes, descriptive statistics and Fisher’s exact tests were used to explore associations between CPV‐2 positivity and host factors (sex, age, and species).

The assessment of risk factors for CPV‐2 positivity in the hunting dog cohort was conducted in two phases. Initially, univariable analysis utilizing chi‐square (*χ* ^2^) or Fisher’s exact tests was performed for all categorical variables, while the Wilcoxon rank‐sum test was employed for numerical variables. Variables exhibiting an association with CPV‐2 positivity (*p*  < 0.20) were considered candidates for the multivariable model.

The final multivariable model utilized penalized logistic regression (Firth’s method) to derive adjusted odds ratios (OR) and 95% CI. This method was specifically chosen to address issues of quasi‐complete separation of data (common with small numbers of positive outcomes in certain categorical levels), thereby ensuring stable estimates and reliable inference for all parameters.

All statistical analyses, including univariable screening and multivariable logistic regression modeling, were performed using R Version 4.5.1 [42]. Specific packages utilized for the analysis included dplyr [42] (as part of the R environment), epiR [43] (for OR and CI calculation), and logistf [44] (for penalized logistic regression).

### 2.6. Relative Risk (RR) Mapping

To assess the spatial distribution of carnivoran *P. carnivoran1* RR, a spatial kernel density estimation was applied using case–control data from hunting dogs (*Canis lupus familiaris*) and sympatric mesocarnivorans sampled in the Valencian Community. Case status was assigned according to molecular results (positive or negative), and coordinates were used as spatial inputs.

RR surfaces were computed using the spatstat package [45] in R Version 4.5.1 [42]. For each group (hunting dogs and mesocarnivorans), intensity functions for cases and controls were estimated with a Gaussian kernel and then contrasted to derive the log‐RR function. The resulting RR values were rasterized and projected to ETRS89/UTM Zone 30N (EPSG:25830) using the terra package [46].

To facilitate interpretation, rasters were masked to the boundaries of the Valencian Community, obtained via the mapSpain package [47]. Maps were exported at 300 dpi resolution with a color scale (viridis palette) capped at a maximum RR value of 6, ensuring comparability between both groups.

## 3. Results and Discussion

### 3.1. *P. carnivoran1* Detection and Prevalence

Of the 546 carnivorans examined, 35 individuals tested positive for *P. carnivoran1* DNA by real‐time PCR, corresponding to an overall prevalence of 6.4%. Among hunting dogs, 27 of 243 samples were positive (11.11%; 95% CI: 7.5%–15.8%), whereas 8 of 303 wild mesocarnivorans yielded detectable viral DNA (2.6%; 95% CI: 1.1%–5.1%). Species‐specific prevalences in wildlife were 1.9% (95% CI: 0.4%–5.4%) in red foxes (3/158), 4.8% (0.6%–16.2%) in beech martens (2/42), 4.4% (0.5%–15.1%) in Eurasian badgers (2/45), and 3.6% (0.1%–18.3%) in common genets (1/28) [48]. The mean Ct values were similar between host groups and generally high, with a mean of 31.94 (range: 23.04–36.66) in dogs and 31.76 (range: 27.38–35.94) in mesocarnivorans. These results suggest relatively low levels of viral shedding at the time of sampling. However, in the absence of histopathological examination of the intestinal mucosa and complete clinical histories for the sampled individuals, these findings should be interpreted with caution. While high Ct values are frequently associated with asymptomatic or subclinical states, they may also represent the early or late stages of an acute infection [49].

Descriptive analyses of wild mesocarnivorans suggested numerically higher infection rates in females, juveniles, and in certain species (beech martens and Eurasian badgers). However, the Fisher’s exact test showed no statistically significant association between positive status and any of the tested factors: sex (*p* = 0.4555), age (*p* = 0.2186), or species (*p* = 0.7307), likely reflecting the low number of positive animals and the resulting limited statistical power. These patterns should therefore be interpreted cautiously as exploratory signals rather than confirmed risk factors.

Most sampled wild carnivorans had died from vehicular collisions, and no animal presented gross lesions suggestive of parvoviral infection. However, in the absence of histopathological analysis and direct PCR from intestinal tissues, it remains possible that subclinical or early‐stage infections were present, although the virus was not the likely cause of their death [4, 17].

As reported in Greene and Decaro [14], PCR methodology has been established as a sensitive means of detecting CPV‐2 in the feces of infected dogs. Although *P. carnivoran1* has been isolated from the feces of clinically healthy domestic and wild felines, difficulty in viral isolation from fecal and intestinal samples of wild species—particularly the red fox—has been noted previously [4]. A key limitation of this study is the reliance on fecal material for *P. carnivoran1* detection. The lack of DNA concentration normalization may have contributed to false negatives, especially in samples with low DNA content or inhibitors.

While our wild carnivoran samples were collected from feces within the large intestine, intestinal tissue has been demonstrated as the most reliable sample to collect from wild animals for *P. carnivoran1* [17]. This is supported by the findings of Petroušková et al. [49], who reported that fecal samples had a significantly lower prevalence than intestinal tissues and rectal swabs. Because our samples consisted exclusively of fecal material—including freshly deposited stools from domestic dogs—the prevalence reported here likely represents a conservative estimate of the true infection burden.

The decision to prioritize fecal material over intestinal tissues for the wild carnivoran cohort was made to ensure methodological consistency across all study groups. While it is well‐established that the intestinal mucosa is a more sensitive matrix for protoparvovirus detection in necropsied animals, a standardized comparison with the domestic dog population required a noninvasive approach. Because collecting intestinal biopsies from the live dog cohort was not ethically or logistically feasible, feces were utilized as the universal matrix.

The inherent variability in sampling strategies and the diversity of molecular methods—ranging from conventional PCR to variant‐specific quantitative PCR (qPCR)—hinder a cohesive comparison across the existing literature [18]. However, we can compare the prevalence found in the current study with other findings in Europe. Our prevalence is very similar to that encountered in Bulgaria, where two out of 57 wild carnivoran intestinal and fecal samples (approximately 4%) were *P. carnivoran1* DNA positive by PCR [50], and in the Iberian Peninsula, as described by Canuti et al. [26] (prevalence of 1.2%) and Miranda et al. [51] (prevalence of 2%).

Conversely, this prevalence is lower than that reported by Petroušková et al. [49] in Slovakia (11%) and Calatayud et al. [18] in spleen samples from Spanish carnivorans (18.3%). It is notably much lower than the prevalence found by Duarte et al. [4] in Portugal (63%), where real‐time PCR detected *P. carnivoran1* DNA in the lymph nodes and intestinal samples of 81 out of 128 carnivorans. This significant disparity is most likely due to the choice of samples—utilizing internal organs versus fecal material—and the inclusion of higher Ct values (ranging from 27.92 to 39.75, average 34.73) in the latter study, which were excluded from the present analysis.

### 3.2. Multivariable Analysis of Risk Factors in Hunting Dogs

Univariable screening identified several candidate risk factors (*p*  < 0.20) for CPV‐2 positivity, including soil substrate, housing management, ownership type, water source, diet, hunting modality, geographic zone, breed, and behavioral traits. Regarding the latter, although scavenging and coprophagy were associated with CPV‐2 status in the initial screening, positive cases were exclusively distributed within the “none” behavioral category. Conversely, clinical signs (diarrhea) and age group showed no significant association with infection and were subsequently excluded from the multivariable analysis (Appendix Table A1). A descriptive evaluation of the positive cohort revealed clear intrapack clustering. Specifically, 20 of the positive hunting dogs belonged to the same large pack, and 5 additional positive cases were aggregated within a second distinct pack. The remaining positive animals (*n* = 2) were individual cases with no shared management frameworks.

Following this screening, a final adjusted model was constructed using penalized logistic regression (Firth’s method). This approach accounted for potential confounding and was finalized after assessing the multicollinearity among the most stable candidate predictors.

The overall multivariable model was highly significant (Likelihood ratio test *p*  < 0.001), indicating a strong predictive relationship between the included factors and CPV‐2 positivity.

The analysis identified the type of diet as the strongest independent risk factor in the final model. While international frameworks and recent reviews emphasize the importance of monitoring environmental pathways, such as wastewater and stagnant water, to predict viral dynamics under a “One Health” approach [12], our multivariable analysis indicates that dietary management remains the primary risk driver in this specific hunting dog population. Compared to the low‐risk diet category (dry food, cooked food), dogs consuming a diet of pure raw meat exhibited a risk that was 34.5 times greater of being positive to CPV‐2 (OR 34.5; CI: 4.38–521.53; *p*  < 0.001), making it the strongest independent factor. Similarly, dogs consuming a mixed diet (dry/cooked food + raw meat) showed a significantly elevated risk (OR = 13.7; 95% CI: 1.26–218.59; *p* = 0.034). All other factors that were highly significant in the univariable screening (including total number of dogs, hunting modality, water source, and dog breed) became nonsignificant in the final adjusted model (*p*  > 0.20).

However, this finding should be interpreted as a strong association rather than a confirmed causal link. In a hunting context, raw meat consumption may act as a direct source of exposure if tissues are contaminated during field processing. Alternatively, this variable may serve as a proxy for broader management practices or environmental exposure specific to certain hunting groups, where the concentration of cases within a few large packs (as evidenced by the 20‐dog and 5‐dog clusters described above) might heavily confound individual‐level factors. In high‐density settings under shared management, specific feeding practices like raw‐meat delivery are often uniform across the entire pack; thus, the massive OR observed for the raw diet likely reflects a cluster‐driven exposure wave within these specific hunting groups rather than an isolated dietary risk. While raw meat‐based diets are recognized sources of various pathogens, the role of meat as a mechanical vehicle for parvoviruses remains a hypothesis as the food items themselves were not tested for viral presence. Furthermore, a limitation of this study is that the specific animal origin of the raw meat (e.g., commercial livestock offal versus field‐dressed wild game) was not recorded. This distinction is relevant, as different sources may carry varying levels of contamination risk or reflect different points of entry for the virus into the domestic dog population. Future research is warranted to investigate the *P. carnivoran1* presence along the raw meat and offal supply chain.

### 3.3. Molecular Characterization and Host‐Strain Patterns

Sequencing of VP2 gene regions was successful for the majority of real‐time PCR‐positive samples and allowed robust typing of viral strains. The nucleotide sequences identified in this study were deposited in GenBank under accession numbers PZ244645 to PZ244671 for the hunting dogs and PZ244672 to PZ244679 for the wild carnivorans. All analyzed hunting dog samples were assigned to the CPV‐2c antigenic variant based on their characteristic VP2 mutation profile. This finding is epidemiologically relevant because CPV‐2c is a predominant field variant associated with clinically significant diseases in dogs across Europe. While most traditional modified live virus vaccines are formulated with CPV‐2 or CPV‐2b strains [52], recent advancements have led to the authorization of recombinant vaccines, such as those incorporating the CPV‐2c 630a strain [53]. However, given that our sampling period for domestic dogs (2021–2022) predates the widespread regional availability of such 2c‐specific formulations, the detected sequences in this study are interpreted as circulating field variants. A limitation of this study is the lack of individual vaccination records for the sampled hunting dogs. This is particularly relevant as vaccination, while highly effective at preventing severe clinical disease, may not completely block subclinical infection or viral shedding upon exposure to contemporary field variants [52]. Consequently, the observed prevalence likely reflects a combination of environmental field pressure and varying levels of vaccine‐induced immunity. Future studies should aim to include precise vaccination histories to further refine the assessment of dietary and environmental risk factors.

Notably, none of the CPV‐2 positive hunting dogs exhibited clinical signs of enteritis at the time of sampling, and their fecal consistency scores were within normal limits. These findings suggest that apparently healthy adult dogs may shed CPV‐2c without overt clinical disease, potentially acting as subclinical carriers and contributing to environmental contamination. However, this interpretation should be treated with caution in the absence of longitudinal monitoring or histopathological confirmation. Similar observations have been reported in other settings, where CPV‐2 DNA has been detected in the feces of asymptomatic owned or free‐roaming dogs at highly variable prevalences. For instance, Ferrara et al. [54] detected the virus in 6.5% of 170 apparently healthy dogs in Italy, and Antiya et al. [55] found that *P. carnivoran1* was the most frequent virus detected in asymptomatic individuals in India (57.73% positivity). Conversely, lower prevalences were noted in studies from Canada in free‐roaming dogs (4.2%; [56]); Germany (2%; [57]); and Spain (0 of the 12 healthy dogs screened; [24]). The present study extends this evidence to hunting dogs within a high‐exposure environment—characterized by the high density of domestic dogs and the endemicity of the virus in the Iberian Peninsula [4, 18, 33]—underlining the importance of apparent subclinical carriage for viral maintenance in domestic populations.

Among the eight wild mesocarnivorans subjected to sequencing, most (6/8) harbored FPV strains (GenBank Accession Numbers PZ244673, PZ244674, PZ244676–PZ244679), while two individuals (one Eurasian badger—Mm21012, Accession Number PZ244675—and one red fox—Vv1613, Accession Number PZ244672) were infected with CPV‐2b. The specific genomic regions and primers used for these amplifications are detailed in Table 1. While FPV sequences lacked CPV‐2‐specific amino acid substitutions, the CPV‐2c sequences from dogs consistently displayed the N426E substitution characteristic of this variant. This mixture of FPV and CPV‐2b in sympatric wild carnivorans is consistent with previous Iberian studies [58, 59], which have documented multiple parvoviral lineages circulating across canids, mustelids, and viverrids, sometimes with identical nucleotide sequences in different host species [18].

Taken together, these molecular data support a complex, multi‐host pathogen system at the wildlife–domestic interface [60]. In this study, wild mesocarnivorans appeared to primarily harbor FPV, while their involvement in the circulation of CPV‐2 lineages was limited to sporadic detections of the CPV‐2b variant. This contrast with the CPV‐2c strains found in hunting dogs suggests that during the study period, these populations were largely infected by distinct parvoviral lineages. However, given the limited sample size, these results should not be interpreted as a definitive exclusion of wildlife as a potential reservoir for CPV‐2c.

### 3.4. Phylogenetic Analysis

While whole‐genome sequencing (WGS) is increasingly the gold standard for viral phylodynamics [8, 60], our molecular characterization relied on the partial sequencing of three distinct VP2 gene regions. This approach was chosen primarily due to the varying DNA quality of field‐collected samples, particularly those from older wildlife carcasses, which often precluded the high‐integrity templates required for long‐range PCR or WGS. We acknowledge that the conceptual assembly of independent fragments carries an inherent risk of describing chimeric sequences in cases of coinfection [61]. To mitigate this, typing results were cross‐validated for consistency across all successfully amplified regions for each individual. While minor amino acid variations were observed in two wildlife strains (see Supporting Information 1: Table S1), these did not affect the overall lineage assignment, and no evidence of coinfection by disparate viral types was detected. Consequently, the consensus sequence from each host was considered to represent the dominant viral population. Furthermore, while residue 426 is a robust marker for antigenic classification, recent evidence emphasizes that this single amino acid substitution does not reflect the true complexity of viral evolution or the monophyletic relationships between strains. As noted by Schirò et al. [8], viruses of the same antigenic type often do not cluster together due to strong selection pressures driving convergent evolution. Therefore, to address the limitations of single‐residue typing at position 426 and to further characterize the detected CPV‐2c and CPV‐2b variants, we extended our analysis to include key lineage‐informative markers within the VP2 gene, specifically at residues 324 and 370 [49]. These positions have been identified as hallmark signatures, among other molecular markers, for distinguishing the traditionally circulating European “Western” strains from the recently emerged “Asian‐like” lineages.

The VP2‐based phylogeny revealed distinct groupings that correspond both to established antigenic designations (CPV‐2a, CPV‐2b, and CPV‐2c) and their geographic lineages. The vast majority of hunting dog strains (26 out of 27) possessed the 324I and 370R mutations, a molecular signature characteristic of the Asian‐like CPV‐2c lineage. This lineage is currently undergoing rapid global expansion and has been documented in Italy and Romania, as well as in Africa and North America [8].

**Figure 2:**
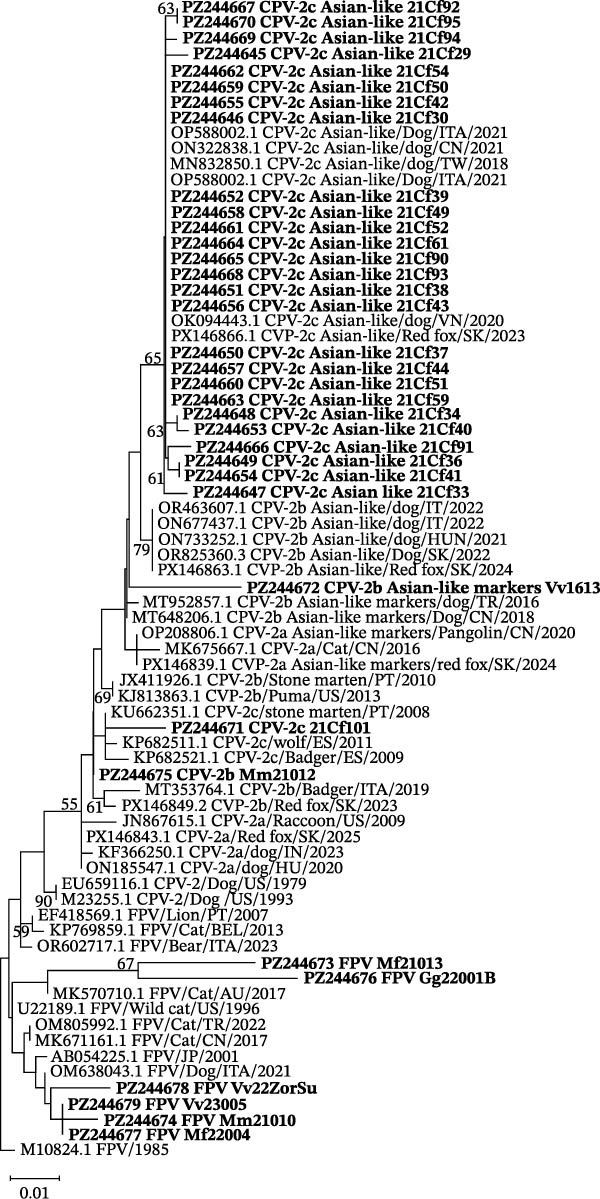
Maximum likelihood phylogenetic tree of *Protoparvovirus carnivoran1* strains based on a ~400 bp alignment of the VP2 gene. The tree illustrates the placement of local strains detected in hunting dogs and wild mesocarnivorans alongside global reference strains. Phylogenetic analysis was inferred using the maximum likelihood method based on the T92 + G substitution model. Values at the nodes represent the percentage of 1000 bootstrap replicates supporting each branch; for clarity, only values ≥50% are shown. The phylogeny was rooted using an ancestral feline panleukopenia virus (FPV) sequence from 1985 (GenBank M10824.1) to establish evolutionary directionality. Sequences generated in this study are shown in bold.

In the phylogenetic tree (Figure 2), all domestic dog samples (with the exception of 21Cf101) aligned within the Asian‐like CPV‐2c cluster, together with contemporary European CPV‐2c strains reported from Italy and Slovakia between 2021 and 2023, as well as reference sequences from China, Vietnam, and Taiwan spanning from 2018 to 2023. In contrast, strain 21Cf101 retained the 324Y and 370Q residues, aligning with the European CPV‐2c phylogroup. This indicates the cocirculation of distinct lineages, albeit with a predominant Asian‐like signature in the domestic population.

Analysis of sequences from wild carnivorans revealed a more complex epidemiological landscape. The red fox strain Vv1613 (PZ244672) and the Eurasian badger strain Mm21012 (PZ244675) were both identified as CPV‐2b variants but occupied distinct phylogenetic positions. The fox‐derived sequence (Vv1613) harboring the 324I mutation clustered within a diverse group characterized by Asian‐like markers, which includes other contemporary wild carnivoran sequences from Europe and Asia, encompassing both CPV‐2a and CPV‐2b backgrounds.

In contrast, the badger‐derived sequence (Mm21012) aligned within the Western cluster, showing close phylogenetic affinity with other European wildlife‐derived CPV‐2b strains, such as those from badgers in Italy and foxes in Slovakia. This broader Western group also encompasses lineages previously reported in North American wildlife and Southwestern Europe, highlighting the wide geographical circulation of this cluster. The remaining parvovirus‐positive wild mesocarnivorans (*n* = 6/8) harbored FPV strains (e.g., Mf22004), which clustered within the FPV clade and are consistent with the circulation of traditional feline‐like strains in the local wildlife cycle. While this broader FPV cluster largely segregates from the domestic dog CPV‐2 interface, its macro‐topography must be interpreted with caution as a localized epidemiological snapshot, given that the reference dataset was optimized for CPV‐2 variant discrimination rather than exhaustive global FPV tracking. Nonetheless, the alignment possesses sufficient resolution to demonstrate that the local wildlife FPV reservoir is not uniform. Instead, clear intracluster divergence is observable, with sequences derived from noncanid wild mesocarnivorans (such as *M. foina*—Mf21013 and *G. genetta*—Gg22001B) forming a highly divergent subgroup characterized by notable horizontal branch lengths and robust node support, standing in contrast to the separate sub‐lineage circulating among local foxes and badgers. To investigate whether these divergent strains represented sequences unique to the study region or had close relatives in public databases, BLAST searches (blastn, NCBI) were performed for both Mf21013 and Gg22001B. The closest database matches retrieved were, among others, MK671161.1 (FPV/Cat/CN/2017) and MK570710.1 (FPV/Cat/AU/2017), showing 96.23% nucleotide identity with Mf21013, and AB054225.1 (FPV/JP/2001), showing 95.69% nucleotide identity with Gg22001B, all of which were incorporated into the phylogenetic tree for direct comparison. The relatively low identity values (~95%–96%) most likely reflect a methodological component inherent to opportunistic wildlife sampling rather than true biological divergence. Within the *P. carnivoran1* species, where FPV and CPV‐2 themselves share ~98%–99% nucleotide identity, a value of ~96% is atypically low and is more consistent with sequencing artifacts introduced by suboptimal DNA quality and limited flanking sequence coverage at fragment extremities—conditions typical of field‐collected wildlife necropsies—than with accelerated biological diversification. These sequences were nonetheless retained in the phylogenetic analysis, as their lineage assignment as FPV remains unambiguous, and they provide genuine epidemiological information regarding FPV circulation in noncanid wild carnivorans in the Valencian Community.

These phylogenetic groupings are further corroborated by a detailed comparative analysis of amino acid substitutions at key VP2 residues provided in Supporting Information 1: Table S1. While most strains displayed the consensus motifs for their respective lineages, minor idiosyncratic polymorphisms were observed in a subset of wildlife sequences, which did not alter the broader phylogenetic clustering.

Similarity identity matrices reinforced these VP2‐based groupings, focusing on representative strains that yielded the highest sequence quality and coverage across the target regions (Supporting Information 2: Table S2). Due to the high sequence homogeneity observed within major host groups, three strains were selected as proxies for the identity analysis: Mm21012 (PZ244675) for CPV‐2b wild carnivorans, 21Cf90 (PZ244665) for CPV‐2c hunting dogs, and Mf22004 (PZ244677) for FPV wild carnivorans. These specific strains provided the most robust templates for comparison against global references.

The badger strain Mm21012 showed 99.8% nucleotide and 100% amino acid similarity to a strain identified in a beech marten in Portugal in 2010 (JX411926.1). Similarly, the hunting dog 21Cf90 exhibited 100% similarity at both nucleotide and amino acid levels to a red fox strain from Slovakia reported in 2023 (PX146866.1). While strains with unique molecular signatures, such as the Western‐like 21Cf101 and the Asian‐marker‐bearing Vv1613, were excluded from the matrix due to lower sequence integrity in specific partial regions, their phylogenetic placement was consistently supported in the phylogenetic analysis (Figure 2). The genetic distinction between the FPV and CPV‐2 clades identified in this work was further confirmed by intergroup identity values. Nucleotide similarity between FPV strains and the CPV‐2 group ranged from 95.1% to 98.6%. Notably, the highest identity value (98.6%) was observed between the FPV strains and the original CPV‐2 strain (EU659116.1). This high degree of similarity is consistent with the well‐documented evolutionary emergence of CPV‐2 from an FPV‐like ancestor in the late 1970s.

It is important to interpret these inferences with caution. While our analysis of VP2‐informative residues (including 324, 370, and 426) provides an improvement over single‐residue typing, these markers still represent a fraction of the viral genome. To achieve a truly comprehensive evolutionary characterization, future studies should incorporate the nonstructural NS1/NS2 genes or whole‐genome data, which would allow for finer‐scale inferences that remain beyond the resolution of VP2‐based datasets alone.

### 3.5. Spatial RR Patterns and Epidemiological Implications

**Figure 3:**
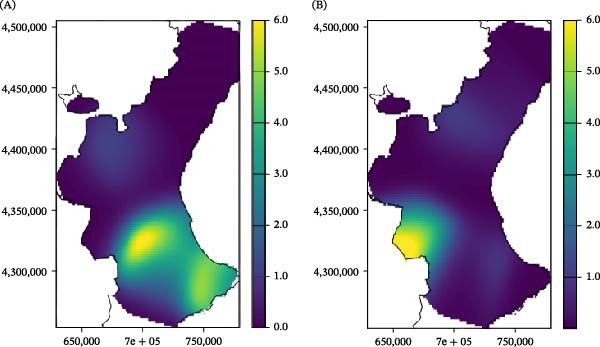
Relative risk (RR) maps of *Protoparvovirus carnivoran1* infection in the Valencian Community, Spain. (A) Estimated RR for wild mesocarnivorans. (B) Estimated RR for hunting dogs, illustrating distinct spatial risk patterns for the domestic dog cohort. RR values were modeled using kernel density estimation and represent the fold‐increase in infection risk compared to the study area average. Values > 1 indicate increased risk, with RR > 4 identifying high‐risk hotspots. The scale in both subfigures is capped at a maximum of 6.0 to facilitate direct comparison. Coordinates are shown in UTM.

RR maps revealed distinct spatial patterns for mesocarnivorans and hunting dogs (Figure 3). In mesocarnivorans, high‐risk areas (RR > 4) were concentrated mainly in the southern part of the Valencian Community, with two visible hotspots: one in the inland area and another along the southern coast. Most of the central and northern regions showed RR values close to 1. In hunting dogs, a single pronounced hotspot was observed in the south‐western inland region, where RR reached the maximum capped value of 6. Outside this focus, most areas exhibited a low to moderate RR ≈ 1–2. The RR map for hunting dogs was generated based on the sampling location (primary residence). Although broader hunting regions were recorded for each individual, these areas lacked the geographic precision required for fine‐scale spatial analysis.

The RR maps suggest that although both host groups are exposed to *P. carnivoran1*, the epidemiological dynamics may differ: mesocarnivorans likely reflect a more diffuse circulation across natural habitats, whereas hunting dogs represent clustered foci that may be associated with specific hunting groups and management practices related to the primary independent risk factor identified—raw meat and offal.

When the spatial and molecular data are considered jointly, an important inconsistency emerges with the simplest hypothesis of direct local spillover from wildlife to dogs. If hunting dogs were acquiring CPV‐2c predominantly from local mesocarnivorans, one would expect a substantial spatial overlap between high‐risk areas in both host groups and detection of the same variant in wildlife. Instead, the dog and mesocarnivoran hotspots only partially coincide, and the CPV‐2c antigenic variant was not detected in any of the sequenced wild individuals, which carried FPV or CPV‐2b in our data.

These observations suggest that CPV‐2c detected in dogs may be maintained through transmission cycles that are at least partially independent of the local wild carnivorans sampled. However, several factors necessitate a cautious interpretation of these findings. First, the spatial points used for the RR maps reflect the primary residence of the dogs at the time of sampling; while hunting areas were recorded, they were often too geographically broad to be utilized for fine‐scale mapping. Consequently, the actual site of viral exposure may differ from the plotted residence. Second, there is a temporal discrepancy between the cohorts: while the mesocarnivoran sampling spanned 2017–2025, the dog samples were collected within a more restricted window (2021–February 2022).

A more plausible working hypothesis is that CPV‐2c is being introduced and maintained in the hunting dog population through routes not fully captured by the current wildlife sampling. Potential explanations include contamination within the raw meat or offal supply chain, the movement of infected dogs between different geographical regions, and the presence of unsampled domestic reservoirs. For instance, feral cats are known reservoirs of *P. carnivoran1* [10, 62] and could play a role in maintaining viral variants at the wildlife–domestic interface that were not detected in our mesocarnivoran cohort. While independent transmission dynamics and variant maintenance in wild populations have been documented in other regions [63], our data cannot definitively confirm or rule out cross‐species transmission interfaces. The failure to detect CPV‐2c in wildlife could be due to the limited wildlife sample size, the lower sensitivity of shedding in feces versus target intestinal tissue, or true ecological segregation. Therefore, more comprehensive sampling and WGS are needed to fully resolve this question. These interpretations remain inferential due to the limited resolution of partial VP2 sequencing and the temporal differences in sampling between the two host groups.

These findings highlight the need to integrate molecular, spatial, and management data when assessing pathogen flow at the wildlife–domestic interface.

## 4. Conclusions

This study identifies a low prevalence of *P. carnivoran1* in wild mesocarnivorans of the Valencian Community, contrasted with a higher prevalence in associated hunting dogs. Notably, the detection of the virus in dogs with normal fecal consistency and the lack of gross lesions in positive wildlife suggest that infected individuals in this region may frequently act as subclinical carriers.

The multivariable model identified a strong association between the consumption of raw meat and an increased risk of CPV‐2 positivity in hunting dogs. This indicates that exposure through potentially contaminated elements of the food supply chain represents a major potential pathway in this domestic cohort. However, a direct causal relationship cannot be definitively established, as the food items themselves were not tested for viral presence. Instead, this powerful dietary association must be interpreted alongside the clear intrapack clustering observed in our descriptive analysis. In high‐density settings under shared management, uniform feeding practices like raw meat delivery heavily overlap with intense horizontal transmission wave dynamics within specific hunting packs. Consequently, raw meat consumption likely acts as a highly effective epidemiological proxy for broader, localized management risks and elevated environmental viral pressure within specific canine aggregates.

Furthermore, the exclusive detection of the “Asian‐like” CPV‐2c variant in dogs—contrasted with the FPV and “Western‐like” CPV‐2b signatures in mesocarnivorans—and the lack of overlapping spatial risk patterns highlight the complexity of multi‐host dynamics at this interface, though a definitive cross‐species transmission pathway cannot be confirmed or ruled out. These findings, complemented by our VP2‐centric phylogenetic analysis, underscore the complex multi‐host dynamics at the wildlife–domestic interface and suggest that local transmission between these populations was not the primary driver of the observed infection patterns during the study period.

Future research incorporating whole‐genome data, alongside investigations into the raw meat and offal supply chain and the role of other reservoirs (e.g., feral cats), will be essential to provide a more robust evolutionary framework and to accurately delineate the lineage structure across hosts.

## Funding

The first author (Francesca Suita) was supported by a predoctoral fellowship from the CEU Cardenal Herrera University (Grant FPI/CEU‐UCH/2022/86).

## Disclosure

Following these AI‐assisted refinements, the authors critically reviewed, verified, and edited all outputs. The authors maintain full accountability for the integrity and accuracy of the final published work. The funding institution had no involvement in the study design, the collection and analysis of data, the interpretation of results, or the preparation and submission of this manuscript.

## Ethics Statement

Ethical approval was not required for this study as all wild carnivoran samples were obtained postmortem. Specifically, American mink specimens were sourced from individuals culled under official invasive species management programs independent of this research. The study adhered to European Union Directive 2010/63/EU and Spanish Royal Decree 53/2013, which exempt nonexperimental clinical or postmortem research from additional permitting. The acquisition of protected species was coordinated by the Generalitat Valenciana under the standing institutional collaboration agreement with CEU Cardenal Herrera University (Ref: 717_18, November 30, 2018).

## Conflicts of Interest

The authors declare no conflicts of interest.

## Supporting Information

Additional supporting information can be found online in the Supporting Information section.

## Supporting information


**Supporting Information 1** Table S1. Amino acid residues at key lineage‐informative positions of the VP2 capsid protein for representative parvovirus isolates identified in this study.

**Supporting Information 2** Table S2. Similarity identity matrix of CPV‐2 and FPV strains, comprising global reference lineages (original CPV‐2, classic CPV‐2a, and Asian‐like 2a, 2b, and 2c markers) alongside representative domestic and wild local fauna isolates (European badger, hunting dog, and stone marten).

## Data Availability

The original data presented in the study are openly available in the GenBank public database under Accession Numbers PZ244645–PZ244679. Additional data supporting the findings of this study are available from the corresponding authors upon reasonable request.

## Appendix A

**Table A1:** Univariable screening of candidate risk factors associated with *Protoparvovirus* positivity in hunting dogs (*p* < 0.20).

Variable	Category	Positives/total	Prevalence (%)	*p*‐Value
Diet	Dry food	1/66	1.5	<0.001 ^∗^
	Mixed	21/107	19.6	
	Others	0/64	0.0	
	Raw meat	5/6	83.3	
Substrate/soil	Concrete	0/122	0.0	<0.001 ^∗^
	Natural soil	27/107	25.2	
Hunting type	Big game	20/120	16.7	<0.001 ^∗^
	Both	6/11	54.5	
	Small game	1/112	0.9	
Breed	Andalusian hound	21/59	35.6	<0.001 ^∗^
	Others	6/184	3.3	
Ownership	Pack	25/136	18.4	<0.001 ^∗^
	Private owner	2/107	1.9	
Housing	Collective	6/127	4.7	<0.001 ^∗^
	Individual	21/116	18.1	
Water source	Natural source	0/17	0.0	0.0049 ^∗^
	Tap water	20/109	18.3	
	Well water	7/114	6.1	
Diarrhea	No	23/217	10.6	0.3728
	Yes	0/13	0.0	
Age group	Adult	23/185	12.4	0.4672
	Geriatric	3/37	8.1	
	Puppy	1/21	4.8	
Other behaviors	None	27/209	12.9	0.0187 ^∗^
	Scavenging/coprophagy	0/34	0.0	

*Note: p*‐Values were calculated using Pearson’s chi‐squared test or Fisher’s exact test as appropriate. Asterisks ( ^∗^) indicate statistical significance (*p* < 0.05). Categories were grouped to ensure statistical robustness and account for typographical variations in the raw data.
