# Supplementary material for: Evaluating Protoparvovirus carnivoran1 Risk in Wild Carnivorans and Hunting Dogs in the Valencian Community, Eastern Spain
Source: Transbound Emerg Dis. 2026 Jul 28;2026:6899927. doi: 10.1155/tbed/6899927 (PMC13408708; doi:10.1155/tbed/6899927)
Supplement: Supplementary file 1 — Supporting Information 1 Table S1. Amino acid residues at key lineage‐informative positions of the VP2 capsid protein for representative parvovirus isolates identified in this study. [file TBED-2026-6899927-s001.docx]

|  | | | **Amino acid residues** | | | | | | | | | | |
| --- | --- | --- | --- | --- | --- | --- | --- | --- | --- | --- | --- | --- | --- |
| **CPV-2/FPV variant** | **Host** | **GenBank Accession Number** | **80** | **87** | **93** | **103** | **297** | **300** | **305** | **323** | **324** | **370** | **426** |
| **“Asian-like” CPV-2b** | Red fox  (Vv1613) | PZ244672 | R | L | N | A | A | G | Y | N | **I** | Q | **D** |
| **CPV-2b** | Eurasian badger (Mm21012) | PZ244675 | R | L | N | A | A | G | Y | N | Y | Q | **D** |
| **“Asian” CPV-2c** | Hunting dogs (26 isolates) | PZ244645 to PZ244670 | R | L | N | A | A | G | Y | N | **I** | **R** | **E** |
| **CPV-2c** | Hunting dog (21Cf101) | PZ244671 | R | L | N | A | A | G | Y | N | Y | Q | **E** |
| **FPV** | Beech marten (Mf21013) | PZ244673 | K | M | K | V | S | - | Y | D | Y | Q | N |
| **FPV** | European badger (Mm21010) | PZ244674 | K | M | K | V | S | A | D | D | Y | Q | N |
| **FPV** | Common genet (Gg22001B) | PZ244676 | K | M | K | V | S | A | D | N | Y | Q | N |
| **FPV** | Beech marten  (Mf22004) | PZ244677 | K | M | K | - | S | A | D | D | Y | Q | N |
| **FPV** | Red fox (Vv22ZorSu) | PZ244678 | K | M | - | - | S | A | D | D | Y | Q | N |
| **FPV** | Red fox  (Vv23005) | PZ244679 | - | - | - | - | S | A | D | D | Y | Q | N |

**Table S1.** Amino acid residues at key lineage-informative positions of the VP2 capsid protein for representative parvovirus isolates identified in this study.
