# Supplementary material for: Evaluating Protoparvovirus carnivoran1 Risk in Wild Carnivorans and Hunting Dogs in the Valencian Community, Eastern Spain
Source: Transbound Emerg Dis. 2026 Jul 28;2026:6899927. doi: 10.1155/tbed/6899927 (PMC13408708; doi:10.1155/tbed/6899927)
Supplement: Supplementary file 2 — Supporting Information 2 Table S2. Similarity identity matrix of CPV‐2 and FPV strains, comprising global reference lineages (original CPV‐2, classic CPV‐2a, and Asian‐like 2a, 2b, and 2c markers) alongside representative domestic and wild local fauna isolates (European badger, hunting dog, and stone marten). [file TBED-2026-6899927-s002.docx]

|  | CPV-2 (EU659116.1) | CVP-2a (JN867615.1) | CPV-2a Asian-like markers (OP208806.1) | CPV-2b (JX411926.1) | CPV-2b Asian-like marker (MT648206.1) | CPV-2b Asian-like (PX146863.1) | CPV-2c (KP682521.1) | CPV-2c Asian-like (PX146866.1) | **CPV-2b Mm21012 (PZ244675)** | **CPV 2c Asian like 21Cf90 (PZ244665)** | **FPV Mf22004 (PZ244677)** |
| --- | --- | --- | --- | --- | --- | --- | --- | --- | --- | --- | --- |
| CPV-2 (EU659116.1) | 100 | 97.241 | 96.552 | 96.552 | 95.862 | 95.172 | 96.552 | 95.172 | 96.552 | 95.172 | 98.621 |
| CVP-2a (JN867615.1) | 98.856 | 100 | 97.931 | 97.931 | 97.241 | 96.552 | 97.931 | 96.552 | 97.931 | 96.552 | 97.241 |
| CPV-2a Asian-like markers (OP208806.1) | 98.627 | 98.856 | 100 | 98.621 | 99.310 | 98.621 | 98.621 | 98.621 | 98.621 | 98.621 | 96.552 |
| CPV-2b (JX411926.1) | 98.627 | 98.856 | 99.085 | 100 | 99.310 | 98.621 | 99.310 | 97.931 | 100 | 97.931 | 96.552 |
| CPV-2b Asian-like marker MT648206.1 | 98.398 | 98.627 | 99.771 | 99.314 | 100 | 99.310 | 98.621 | 98.621 | 99.310 | 98.621 | 95.862 |
| CPV-2b Asian-like (PX146863.1) | 97.941 | 98.169 | 99.314 | 98.856 | 99.542 | 100 | 97.931 | 99.310 | 98.621 | 99.310 | 95.172 |
| CPV-2c (KP682521.1) | 98.169 | 98.398 | 98.627 | 99.085 | 98.856 | 98.398 | 100 | 98.621 | 99.310 | 98.621 | 96.552 |
| CPV-2c Asian-like (PX146866.1) | 97.712 | 97.941 | 99.085 | 98.627 | 99.314 | 99.314 | 98.627 | 100 | 97.931 | 100 | 95.172 |
| **CPV-2b Mm21012 (PZ244675)** | 98.856 | 99.085 | 99.314 | 99.771 | 99.542 | 99.085 | 99.314 | 98.856 | 100 | 97.931 | 96.552 |
| **CPV 2c Asian like 21Cf90 (PZ244665)** | 97.712 | 97.941 | 99.085 | 98.627 | 99.314 | 99.314 | 98.627 | 100 | 98.856 | 100 | 96.568 |
| **FPV Mf22004 (PZ244677)** | 98.398 | 97.712 | 97.483 | 97.483 | 97.254 | 96.796 | 97.025 | 96.568 | 97.712 | 95.172 | 100 |

**Table S2. Similarity identity matrix of CPV-2 and FPV Strains**. The matrix comprises several groups of sequences. Regarding global references, it includes the original CPV-2 (EU659116.1), the classic CPV-2a (JN867615.1), and various Asian-like markers across the 2a, 2b, and 2c lineages (OP208806.1, MT648206.1, and PX146866.1, respectively). Furthermore, the study presents representative isolates from local fauna: CPV-2b Mm21012 (PZ244675) from a European badger, representing circulating wild strains; CPV-2c Asian-like 21Cf90 (PZ244665) from a hunting dog, representing the domestic population; and FPV Mf22004 (PZ244677) from a stone marten, which serves as a proxy for the five identical FPV isolates identified in this work.
